# Baseline Characteristics and Outcomes for the 4F Pilot for Persons Aging with HIV

**DOI:** 10.1093/geroni/igaf122.2674

**Published:** 2025-12-31

**Authors:** Julie Womack, Chloe Johnson, Evelyn Hsieh, Richard Marottoli, Wynnett Stewart, Michael Virata, Lydia Aoun-Barakat

**Affiliations:** Yale University School of Nursing, Orange, Connecticut, United States; Yale School of Medicine, New Haven, Connecticut, United States; Yale School of Medicine, New Haven, Connecticut, United States; Yale University School of Medicine, New Haven, Connecticut, United States; Yale New Haven Healthcare System, New Haven, Connecticut, United States; Yale School of Medicine, New Haven, Connecticut, United States; Yale School of Medicine, New Haven, Connecticut, United States

## Abstract

**Background:**

More than half of people living with HIV in the US are 50+ years of age. HIV clinicians must be comfortable assessing for and managing conditions associated with aging.

**Methods:**

We trained 11 clinicians at an academic HIV ambulatory care center to assess and prevent the 4F’s (polypharmacy, fragility fractures, falls) among their patients 50+ years old. We share baseline patient data and results from the HIV clinicians’ knowledge, attitudes, and practices baseline assessment.

**Results:**

125 patients were enrolled. Mean age was 63±6 years, 49% were women, 48% identified as Black. 30% were current smokers, and 30% reported ongoing marijuana use. The mean number of medications prescribed was 10±5. However, 25% experienced hyperpolypharmacy (taking 15+ medications), and 66% were prescribed potentially inappropriate medications (PIMs). Mean FRAX scores indicated low 10-year risk for fragility fractures (major osteoporotic fracture: 8.71%±6.95%; hip fracture 2.11%±3.58%). Using our serious fall risk assessment tool, we found that 27% of women and 24% of men were at high risk for a serious fall within the next 6 months. Among the clinicians participating in the study, 73% performed medication reconciliation at least once a year. 55% reported asking about falls only if the patient brought it up, and 36% had only asked their patients once about falls. 18% had never assessed their patients’ fracture risk.

**Conclusions:**

Polypharmacy, high medication count, and PIMs are key risk factors for adverse outcomes among older adults. Deprescribing and fall and fracture prevention are key foci for future interventions.

